# Salt Chemistry Regulates Habitable Microenvironments in Brinicles: Implications for Icy Ocean Worlds

**DOI:** 10.21203/rs.3.rs-10526536/v1

**Published:** 2026-08-13

**Authors:** Madeline Garner, Marika Feduschak, Scott Perl, Heidi Smith, Christine Foreman

**Affiliations:** Montana State University; Montana State University; Blue Marble Space Institute of Science; Montana State University; Montana State University

**Keywords:** Brinicles, ice, seawater, liquid fraction, habitability, brine

## Abstract

Brinicles are dynamically growing ice structures that have been proposed as chemically active systems on Ocean Worlds, potentially serving as habitable niches for life. However, experimental studies of their internal microstructures and implications for habitability remain limited. This study examined how brine composition influences the formation of liquid habitats within brinicles and how these differences impact the transport and distribution of cellular proxies. Flow cytometry and cryo-Raman spectroscopy were used to quantify the spatial distribution of cell surrogates, liquid brine, and hydrated salts across the radial and vertical axes of brinicles grown under two salt compositions (NaCl and CaCl_2_). Our findings demonstrate fundamental differences in brinicle microstructure between the two salt compositions. CaCl_2_ grown brinicles exhibited confined localization of liquid brine towards the inner channel, whereas NaCl brinicles showed a more homogeneous distribution of the liquid fraction. Ultimately, these differences provide a physical mechanism for cellular surrogate localization in the ice. CaCl_2_ brinicles resulted in the entrapment of microspheres near the inner hole while the NaCl brinicles produced uniform distribution throughout the ice. These results demonstrate that brine chemistry controls habitat structure and biological transport within brinicles, highlighting the importance of ocean composition when assessing habitability on Ocean Worlds.

## Introduction

Brinicles are unique cryobrine systems that have been recorded in the polar regions of Earth [2–4]. Unlike homogeneous ice structures, these systems do not form through a uniform freezing process, but rather an ongoing phase change. Brinicles nucleate as concentrated brine, trapped within the sea ice drains into the underlying ocean [2, 5]. This supercooled highly saline solution triggers rapid freezing of the surrounding seawater upon contact, creating a hollow tube of ice that descends from the sea-ice interface [2, 6]. Within the developing structure, competing thermodynamic freeze-thaw processes govern the growth of the brinicle. The outer wall thickens as heat is transferred from the warmer ocean water to the supercooled internal brine solution, while the interior wall melts as the internal brine column increases in temperature. The resulting meltwater cools and dilutes the internal brine column, feeding back into the cycle of phase change and heat transfer [5]. The coupled processes of heat transfer and varied brine dynamics drive a continuous state of phase change, creating a dynamic environment where the ice is neither completely frozen nor fully melted, but sustained by the gradients generated during growth [7].

Because of this dynamic growth process, brinicle systems have been proposed as chemically active environments that could exist on Europa [8–11]. Early theoretical studies posited that these structures may function as icy world chemical gardens, capable of providing energy gradients that could support prebiotic chemistry [9, 10, 12]. Following this, investigators modeled brinicle formation under Ocean World relevant conditions, predicting that these structures could form and persist beneath Europa’s ice shell for timescales ranging from hours to years, depending on ice shell thickness [9, 13].

Despite brinicles being characterized by their freeze-thaw growth driven processes, many models assume the brinicle ice forms as a simplified ice phase, even though these structures are widely recognized to exist in a ‘mushy’ or mixed-phase state [2, 14, 15]. Recent modeling work has begun to incorporate mixed-phase behavior, predicting the development of a ‘mushy layer’ region surrounding the descending brine plume [7]. However, these models do not resolve differences in brine inclusion volume, connectivity or spatial distribution within the ice matrix. Ionic composition and salinity have previously been shown to impact physical controls of the brinicle [5, 7, 11]. Specifically, brine composition influences key parameters such as freezing point depression, brine density, and viscosity [16]. These differences have been shown to influence brinicle growth rate and morphology [11]. Nevertheless, whether these salt-driven macrostructural changes also determine microstructural variations in brine distribution, and whether such microstructural variation in turn influences the localization of cells, remains unstudied.

The presence, abundance, and distribution of liquid brine within sea ice microstructures represents one of the physical mechanisms that define habitability within these systems [17–19]. During ice formation, salts are rejected from the growing ice lattice, causing the solutes to become concentrated within networks of brine inclusions and veins [17, 18] (Fig. 1). The resulting distribution and connectivity of these liquid brine networks strongly impacts nutrient transport, microbial access to resources, and the overall habitability of these icy systems [17–19]. As a result, the liquid fraction in ice has been proposed as an important proxy of potential habitable space, both in terrestrial sea-ice environments and in the context of Ocean Worlds [19].

Brinicles represent localized regions of brine drainage within sea ice systems. Because the liquid fraction and microstructural brine distribution has not been systematically evaluated in brinicles, their habitability potential remains poorly constrained. Here we address this knowledge gap by quantifying the effects of salt composition on liquid fraction, brine distribution, and the incorporation and transport of microbial cell analogs. Our findings demonstrate that salt composition influences not only the macroscale growth and morphology of brinicle systems, but also the microscale distribution of liquid brine and biological constituents, directly impacting the habitable space within.

## Methods

### Brinicle Growth

The brinicle growth procedure followed those detailed in [11]. Briefly, brinicles were grown for two hours in a ten-gallon tank inside the Subzero Laboratory at Montana State University (Temperature range: −68°C to 60°C, ± 1°C). The room temperature was maintained at a temperature of ~ −20°C with a heat table under the tank set to 6°C. The water temperature was maintained at approximately 0°C. Brine solution was pumped in at a rate of 2.8 mL/min using a peristaltic pump (Cole Parmer Instruments Pump Motor 6–600 RPM, MASTERFLEX 06402–14 14mm Norprene Precision Pump Tubing). Brinicles were grown under two salt treatments: 200 PPT NaCl and 200 PPT CaCl_2_, each prepared with 10^6^ florescent microspheres per liter.

## Flow Cytometry and Microsphere Imaging

### Fluorescent Microspheres

Fluorescent microspheres were used to model transport and localization of cells within the brinicle [20]. Microsphere concentration of 10^6^ florescent microspheres per liter was chosen as conservative estimate of lower limits of microbial abundance recorded in sea ice systems [21]. The microspheres chosen were 2.0 um, yellow-green, fluorescent, 505/515, Molecular Probes. Microsphere size was selected to emulate bacterial cells that are known to partition into ice veins [22]. The carboxylate-modified surface of the microspheres results in a negative charge, which mimics the bacterial cell surface charge [20]. All microsphere suspensions were stored in the dark at 4°C and vortexed immediately before use to prevent settling.

## Fluorescent Microsphere Imaging

Image of ice grains was performed on a Nikon Eclipse E800 with a Zeiss LD Achroplan 20× objective. Sample consisted of deionized water at − 20°C where 1.0 μm yellow-green, fluorescent carboxylate-modified microspheres (Invitrogen F8823) can be seen localizing in liquid filled veins.

## Flow Cytometry Analysis

Five replicate brinicles were grown at each of the two salt compositions for flow cytometry analysis. Following their growth, brinicles were sectioned into four segments proportional to their max length. The first quarter (tip section, 0–25% of the brinicle length) was excluded from analysis due to insufficient meltwater volume. The sections sampled were labeled L3 (25–50%), L2 (50–75%), and L1 (75–100%), where L1 is the segment closest to the base and L3 is the section nearest to the tip (Fig. 2a). Additionally, brine drain outflow was collected for each of the replicates.

Fluorescent microsphere concentrations in brinicle meltwater samples were quantified using a flow cytometer, ZE5 Cell Analyzer with Everest Software v3.2 (Bio-Rad Laboratories, Hercules, CA). A 200 μL meltwater volume was loaded per well, with 50 μL used per sample run at a flow rate of 1.5 μL/s. Samples were agitated for five seconds prior to analysis to minimize microsphere settling. Microspheres were detected using the 488-nm excitation laser with GFP emission filters (505/515 nm). Quantification and gating were determined using a calibration series of microspheres spiked into brinicle meltwater across a range of known concentrations (**Figure S1**). The concentration of microspheres was determined using the calibration standards and is reported as microspheres mL^−1^. Three technical replicates were run for each meltwater sample, yielding 15 replicates per section and salt treatment.

### Cryo-Raman Spectroscopy

Three brinicles with each salt composition were grown and analyzed using cryo-Raman spectroscopy. Following extraction from the tank, each brinicle was flash frozen in liquid nitrogen to capture the brine within the ice [23]. Brinicles were sectioned at the same vertical locations used for the flow cytometry analysis described above (Fig. 2), mounted on an aluminum slide, and thinned down using a microtome operated in a cold room with ambient temperatures of −10°C and − 20°C for the NaCl and CaCl_2_ treatments, respectively. Raman intensity maps were generated with a Horiba Scientific LabRAM Evolution Confocal Raman microscope system using either a 50x (Olympus SLMPLN 50x, NA = 0.35, WD = 18 mm) or 10x (Olympus MPLN 10x, NA = 0.25, WD = 10.6 mm) long working distance objective. Sample slides were housed within a Linkam LTS420 thermal stage cooled by a liquid nitrogen dewar attachment with a temperature stability < 0.01°C and cooled by a liquid nitrogen dewar attachment. Spectra were acquired using a 532 nm laser (450 mW nominal power) at 25% of maximum power (112.5 mW) from 1000 to 3800 cm^−1^ for 0.3 s with a grating of 300 lines/mm. Map size was dependent on the specific section and determined by measuring radially from the inner edge of the hollow tube to the outer edge of the brinicle. Each map was 9–12 μm steps wide and up to 14,000 μm in length (approximately the size of the viewing window of the Linkam thermal stage), resulting in 3,700–10,200 pixels per map, each of which took up to > 1 hr to acquire.

To analyze the hydrated salt distribution, samples were mapped below their eutectic temperatures, at −30°C and − 100°C for the NaCl and CaCl_2_ brinicles, respectively. Three adjacent replicate maps were taken along the radial transect of the section at each temperature to increase statistical robustness (Map 1–3; Fig. 2B). Samples were then warmed up at a rate of 2°C/min up to their eutectic temperature, at which point the rate was changed to 1°C/min up to −7.5°C, where samples were equilibrated for 10 min prior to analysis. This allowed monitoring of samples via video camera to confirm that melting was not excessive nor premature, in order to preserve the ice sample, while also minimizing total experiment time. The final temperature (−7.5°C) was chosen as the midpoint between the brinicle tank water temperature (~ 0°C) and the brine solution temperature (~ −15°C). Another set of three adjacent maps was taken for each section at this final temperature. This process resulted in 6 maps per brinicle section, with 9 replicates for each combination of vertical section, salt composition, and temperature treatment. Figure 3A–D depict representative maps of each measurement condition (CaCl_2_ at −7.5°C, CaCl_2_ −100°C, NaCl − 30°C, and NaCl − 7.5°C) overlaid on a microscope image of one brinicle section (rescaled to show detail). During data analysis, these transects were divided into three equal sections along the length to represent inner, middle, and outer sections used for radial distribution calculations.

## Compositional Analysis

Before any spectral peak analysis was performed, an intensity threshold of 60 arbitrary units (a.u.) was applied to each map in order to remove pixels of insufficient quality. In certain cases, map transects crossed neighboring brinicle holes or fractures, resulting in noisy, low-intensity spectra due to laser interaction with the exposed aluminum slide. Additionally, surface roughness across the brinicle sections occasionally prevented optimal laser focusing, resulting in spectra with insufficient signal quality for analysis. Most maps were of sufficiently high quality that no pixels were excluded; however, maps containing large surface depressions required the removal of a substantial number of pixels.

Overall, following spectral preprocessing only 1.8% of collected pixels were discarded. For all maps taken at −7.5°C, the surface area fraction of liquid was calculated by creating a binary map of brine and ice pixels (Fig. 3). Ice was identified using the OH-stretching band (OH-sb) of water molecules observed between 2800–3600 cm^− 1^. Within this range, a low wavenumber peak (~ 3000–3200 cm^− 1^) corresponds to symmetric stretching of covalent OH bonds while a higher wavenumber peak (~ 3350–3500 cm^− 1^) corresponds to asymmetric stretching [24, 25]. The relative strength of each mode in a sample determines the overall shape of the peak, which can be used to calculate the bulk phase via a ratio known as the disorder parameter [26, 27]:

1 $$S_{D}=\frac{\Delta \;I_{\text{asym}}}{\Delta \;I_{sym}}$$

where ΔI_sym_ is the area under the curve for the symmetric region integrated from 3080–3200 cm^− 1^, and ΔI_asym_ is the corresponding asymmetric region integrated from 3300–3420 cm^− 1^ [26]. Since ice is a more ordered system, imposed by its crystalline lattice, symmetric vibrations are more common, while in liquid samples, asymmetric vibrations dominate. The above integration regions allow for a quantitative bulk phase identifier where S_D_ > 1 indicates water and S_D_ < 1 indicates ice. In these measurements, the presence of NaCl and CaCl_2_ in liquid solution does not change the vibrational environment significantly, and thus, brine is also detectable using S_D_ analysis [27].

For maps collected below the eutectic temperature of NaCl and CaCl_2_, the hydrated salt fraction was determined by calculating the percentage of pixels classified as hydrated salt relative to the combined ice and salt pixels. Unlike liquid NaCl and CaCl_2_ solutions, solid hydrohalite (NaCl.2H_2_O) and antarcticite (CaCl_2_ · 6H_2_O) have characteristic Raman spectral features allowing for spatially resolved differentiation from ice [27, 28]. Both the hydrohalite and antarcticite produce an ice peak at ~ 3135 cm^− 1^ but have higher wavenumber features that are specific to each individual hydrate. Hydrohalite has two main peaks at ~ 3425 cm^− 1^ and 3539 cm^− 1^ (**Figure S2**) [28, 29], while the primary peak for CaCl_2_ hydrates is at ~ 3430 cm^− 1^ [30]. The two salt hydrates exhibited similar Raman spectral profiles, especially given the short acquisition times and wide spectral grating used, so the same salt identification method could be applied to both brinicle compositions. As in the ice/liquid classification approach, integration regions were used, centered on the characteristic the ice and salt peaks: 3110–3170 cm^− 1^ (ΔI_ice_) and 3380–3440 cm^− 1^ (ΔI_salt_). A third range just before the sharp salt peak from 3310–3370 cm^− 1^ (ΔI_val_) was chosen as a “valley” region for comparison (**Figure S2**). Using an approach similar to previous identification schemes [29], each pixel was classified as salt when ΔIice > ΔIval and ΔIsalt > ΔIval. Subsequently, pixels that failed this criterion were marked as ice if the S_D_ was < 1. Pixels that were identified as neither ice nor hydrate were set aside for manual classification, which was rare; occurring only 1.3% of the time (see Supporting Information). Pixels with an intensity ≥ 500 a.u. at 1000 cm^− 1^ were classified as containing fluorescent microspheres, providing a proxy for the localization of cell analogs within the brinicle structure (Fig. 3). To verify that the fluorescent signal originated from the microspheres, ice standards with and without microspheres were analyzed. This verification was performed out of an abundance of caution, as ice, brine, and salt hydrates have previously not exhibited fluorescence under these conditions. Fluorescence assessment was completed after thresholding but prior to other compositional analyses, since this order determined whether a pixel’s fluorescence was low enough to be reliably classified as ice, brine, or salt. Because the microspheres used in this study have a maximum emission wavelength of 515 nm, the fluorescence peak is expected to occur to the left of the 0 cm^− 1^ Raman shift position (corresponding to 532 nm), and to decrease in intensity towards the higher wavenumbers. In some cases, the fluorescence contribution was diminished enough at the range of our measurement peaks that they could still be used for Raman compositional analysis.

### Statistical Analysis

Statistical analyses were performed using Minitab Statistical software (version 22.3.1.0.; Minitab LLC, State College, PA, USA). Data was tested for assumptions of normality and homogeneity of variance. A linear mixed model was used for analysis of data. Tukey post hoc comparisons was utilized to identify pairwise differences after a significant main effect or interaction was observed. Data analysis and visualization were performed using MATLAB 2024b (The MathWorks, Inc., Natick, MA, USA). Claude Opus 4.6 and Sonnet 4.6 (Anthropic, San Francisco, CA, USA) were used to assist in modifying MATLAB code for figure formatting and visualization. Additionally, they were used to create a MATLAB app which allowed for step-by-step data analysis and real time visualization of discarded, fluorescent and ice/brine or ice/hydrate pixels. The criteria used to analyze pixels were not developed by AI, and all subsequent code was reviewed and often edited manually.

## Results

### Fluorescence Cell Analog Bulk Microsphere Distribution

To examine cell analog retention and brine microenvironments within brinicles, complementary bulk and microscale analyses were performed. Flow cytometry was used to quantify bulk microsphere distribution along the length of each brinicle and assess vertical retention across the brinicle (Fig. 4). Cryo-Raman was utilized for the quantification of liquid fraction, hydrated salt fraction, and fluorescence fraction across both the brinicles vertical and radial axes. For each response variable (i.e. bulk microsphere measurement, liquid fraction, hydrated salt fraction, and fluorescence fraction), linear mixed-effects models were used to evaluate the effects of location, brine composition, and their interaction. Tukey post hoc comparisons were used to identify differences among groups where appropriate.

To determine whether brinicle growth physics alone controls bulk microsphere transportation, the concentrations were enumerated across all four vertical locations with both compositions averaged together (Fig. 4). This analysis found that location within the brinicle significantly impacted the abundance of microspheres (p < 0.001). Tukey analysis revealed that the brine drain output was the driver of the section level significance, with the drainage point being significantly higher in microsphere abundance when compared to all other sections (p = 0.001–0.015) (Fig. 4). While a trend existed, there was no significant difference found between microsphere distribution across the remaining three brinicle ice sections (L1-base, L2-mid, and L3-tip). These results demonstrate that the microsphere concentration is highest at the brine exit point for both salt treatment types, with no statistically significant vertical gradient within the ice.

We then examined whether salt composition impacted microsphere retention across the entire brinicle. The analysis found that NaCl and CaCl_2_ showed no significant difference between the mean log transformed microsphere concentrations. Across all locations, the average value for the NaCl treatment was 69 microspheres/mL while the average for CaCl_2_ was 10^6^ microspheres/mL. These results demonstrate that brine chemistry alone does not significantly modulate microsphere retention.

The final analysis tested whether location and composition interacted to influence the spatial pattern of microsphere localization. A significant difference in microsphere abundance was seen when considering the interactions of both composition and location (p < 0.038), reflecting a divergence in trends between the two compositions. The CaCl_2_ treatment showed a continuous increase in microsphere concentration towards the tip of the brinicle, while the NaCl treatment remained relatively uniform in localization (Fig. 4). Tukey pairwise comparisons found no significant difference between any two individual locations and compositions. However, the significant interaction reveals variations in distribution trends rather than isolated differences.

### Liquid Fraction Distribution

The vertical distribution within and between salt compositions was analyzed to determine whether salt chemistry impacts the retention of the liquid fraction. Vertical sectioning alone significantly impacted liquid fraction (p < 0.001). Across all sections, NaCl grown brinicles had a significantly higher liquid fraction (13.5%) when compared to CaCl_2_ brinicles (8.5%) (p = 0.033). In addition, the interaction between brine chemistry and vertical section significantly impacted the liquid fraction (p = 0.001). NaCl brinicles demonstrated strong vertical patterning, with the tip containing significantly more liquid than the mid and base sections (p = 0.001) (Fig. 5C). In contrast, CaCl_2_ grown brinicles had the largest accumulation of liquid fraction at its midpoints, with a significant difference between base and mid (p = 0.021) (Fig. 5C). When both salt compositions were pooled together, Tukey analysis found a significant difference between the base and tip of the brinicles (p < 0.001), with the tip containing more liquid.

To determine whether liquid fraction varied radially, we analyzed the cross-sectional distribution both within each composition and between the two salt treatments. While the pattern of distribution significantly varied between salt treatments (p < 0.001), the total liquid fraction across all three sections did not (p = 0.578) (Fig. 5A). Liquid fraction was significantly impacted by the interaction of brine chemistry and radial location (p < 0.001). The CaCl_2_ grown brinicles exhibited a steep increase in liquid fraction from the outer edge towards the inner hole, with the inside region being significantly different from the middle and outer edges (p = 0.034 and p < 0.001 respectively). In contrast, NaCl brinicles exhibited the opposite distribution with a significant difference between the inside and outside region (p = 0.034) (Fig. 5A). Tukey analysis of the combined compositions revealed that L3R2 (tip middle region) had the highest average liquid fraction for both compositions. This suggests a convergent point for liquid fraction accumulation at vertical and radial gradients.

### Hydrated Salt Fraction Distribution

We analyzed vertical hydrated salt distribution within each composition and between salt treatments to investigate whether salt type impacted vertical retention. The vertical hydrated salt fraction distribution was independent of salt composition (p = 0.314). However, the vertical section was significant when pooling salt compositions together (p < 0.001). The interaction between vertical section and brine chemistry was not significant (p = 0.727), indicating that growth physics alone affect the vertical distribution of the salt hydrate within the brinicles. For both salts, the mid-section acted as an intermediate in hydrated salt fraction (Fig. 5D). Both salt treatments shared a pattern of brine and salt localization away from the base of the brinicle.

Next, the hydrated salt localization was examined independently and comparatively across salt treatments to determine the impact composition has on radial distribution. Radial position alone significantly impacted hydrated salt fraction (p < 0.001). In contrast, composition alone was not significant (p = 0.314). The interaction between salt composition and radial region had a significant impact on hydrated salt fraction (p < 0.001). The CaCl_2_ brinicles had a steep decline in hydrated salts from the inner hole to the outer edge, with R1 being significantly higher than the middle region (p < 0.001) and the outer regions (p < 0.001). For NaCl brinicles, the hydrated salt was evenly dispersed across the radial transect, with no significant difference observed between groups (Fig. 5B). For both treatments, the hydrated salt patterns mirror the distribution seen in the liquid fractions (Fig. 5A **and** B).

### Fluorescence Fraction Distribution

To assess whether salt composition influences fluorescence distribution (cell analog concentration), we quantified the fluorescence fraction within each vertical section of the brinicles and compared these distributions between salt compositions. Composition alone significantly impacted fluorescence fraction (p < 0.001) while the vertical location alone was not influential (p = 0.132). However, the interaction between vertical position and brine chemistry was significant (p = 0.025). Consistent with the trends observed for hydrated salt and brine fractions, the CaCl_2_ brinicles exhibited an increase in fluorescence from the base to the tip (Fig. 6B). Specifically, the base sections of the CaCl_2_ brinicles contained significantly lower fluorescence than the tip section (p = 0.017). In contrast, the NaCl brinicles showed no evidence of a vertical fluorescence trend, with fluorescence fractions remaining statistically similar across all sections (Fig. 6B).

Following this, fluorescence fraction patterns across the brinicles’ radial axes were assessed. Fluorescence fraction was significantly impacted by all three factors: radial region alone (p < 0.001), composition alone (p < 0.001), and their interaction (p < 0.001). The CaCl_2_ treatments produced significantly higher fluorescence fractions than the NaCl group (p < 0.001). This bulk difference is primarily driven by the inner hole dominated distribution of CaCl_2_ where R1 has significantly higher fluorescence than all other radial positions (p < 0.001) (Fig. 6A). However, the NaCl group had a uniform localization of fluorescence fraction, resulting in no significant difference between sections (Fig. 6A).

### Mechanisms Underlying the Coupling Between Fluorescence and Brine Microenvironments

We examined if the liquid fraction could predict hydrated salt localization. Despite the observed compositionally dependent radial distributions, at the individual section level, the liquid fraction significantly predicted the hydrated salt fraction (p < 0.001). On average, for every percentage point increase in liquid fraction, hydrated salt fraction increased by 0.683 percentage points regardless of the composition being tested. The interaction with composition had no significant impact, demonstrating that sections with higher liquid fraction consistently contained more hydrated salt, regardless of brine chemistry. While any liquid phase present at −7.5°C must necessarily contain solutes that remained unfrozen, the co-localization of hydrated salt and liquid fractions confirmed that the liquid phase should be classified as brine rather than pure water.

Having established the spatial distribution of fluorescence in the brinicles, we next evaluated whether the liquid fraction was able to predict fluorescence (Fig. 7). Our analysis revealed that the liquid fraction was a significant predictor of the fluorescence fraction (p < 0.001). Furthermore, the interaction between composition and liquid fraction revealed significantly different slopes (p < 0.001), indicating that the relationship differed between brine chemistries. In the CaCl_2_ treatment, for every percentage point increase in liquid fraction, fluorescence increased by 0.159 percentage points. In contrast, NaCl grown brinicles exhibited a weaker response, with fluorescence fraction increasing only 0.081 percentage points for every percentage point increase in liquid fraction (Fig. 7A). Overall, the liquid fraction explained 49.88% of the observed variation in fluorescence fractions across both salt compositions.

Following this, we examined whether the hydrated salt fraction significantly predicted fluorescence. The hydrated salt fraction was found to significantly predict fluorescence (p < 0.001), with a pooled slope for both salt compositions of + 0.052 (95% CI: 0.028–0.077). However, there was no significant interaction between salt composition and fluorescence (p = 0.080). While the fluorescence co-localized with the hydrated salts, this association is secondary to that observed in the liquid fraction. The hydrated salt fraction explained far less of the variation in fluorescence than that of the liquid fraction, accounting for only 21.37% of the variance

Collectively, these analyses revealed that liquid fraction, hydrated salt fraction, and fluorescence localization were significantly influenced by brine chemistry and spatial positioning within the brinicle. Flow cytometer analyses demonstrated that the bulk microsphere concentration was highest at the drainage point, independent of chemistry. Regions with increased liquid fraction were strongly associated with increased hydrated salt and fluorescence fraction. Brinicles grown in CaCl_2_ solution showed concentrated localization of all three measurements at the inner radial hole. In contrast, NaCl exhibited uniform distribution across all sections. These results demonstrated that when analyzed by section, liquid fraction was the strongest predictor of fluorescence (R^2^ = 49.88%). This association was approximately twice as strong in CaCl_2_ when compared to NaCl (slopes 0.159 vs. 0.081, interaction p < 0.001). Together, these findings demonstrate that the liquid brine environments within brinicles are the primary determinants for microsphere retention and localization within the ice.

## Discussion

Although brine chemistry had no significant impact on bulk microsphere abundance, microstructural analysis revealed compositionally modulated spatial differences within the ice. Salt composition impacted both vertical and radial localization of liquid and fluorescence fraction. These spatial patterns shaped microenvironment connectivity and proximity to the central brine column. As a result, the distribution of liquid brine provides a physical mechanism for the localization of microspheres (cellular analogs) within the ice.

The brinicle drainage retained the highest abundance of microspheres (Fig. 4), while the individual brinicle sections showed no significant difference in bulk microsphere retention. Additionally, brine composition did not significantly impact the microsphere quantity. These results suggest that regardless of brine chemistry, the bulk particle abundance was concentrated at the brine drainage region. This behavior mirrors sea ice where particle and brine solutions are transported through high permeability regions, with material released primarily at the base of the ice [31]. Imaging of brine channels within sea ice has shown that they form penetrating streamers that release into the surrounding water [32]. Brinicles have previously been modeled as conduits through which brine and particles are transported toward a terminal export region [14, 15]. Our experimental results demonstrate that bulk microsphere abundance at the drain output is consistent with previous models, suggesting brinicles function analogously to sea ice brine channels where material is exported to a terminal region.

A significant interaction between location and brine composition was observed for the bulk distribution of microspheres in the brinicles. While brinicles grown in CaCl_2_ showed a gradual increase in microsphere concentration towards the tip, NaCl derived brinicles remained relatively uniform throughout their structure (Fig. 4). These trends reflect subtle differences rather than composition-regulated bulk retention. Microstructural analysis of the ice matrix revealed that these trends were driven by compositionally dependent differences in the liquid fraction.

In sea ice systems, transport alone does not determine habitability [33–35]. The transport of brine exists at two fundamentally different scales: the macroscale, which is gravity driven and controls the bulk material exchange [34] and the microscale, where the movement of brine exists within intergranular veins and brine inclusions [36–38]. While transport is critical for the replenishment of nutrients at both scales, habitats that remain connected to these transport networks are favored more than those in isolated pockets [19, 34, 35]. Previous work has shown that hydrated salt and liquid fraction within the ice regulate where and how these microenvironments can exist [19, 39, 40]. Consequently, resolving the microscale spatial distribution of the liquid fraction within brinicle ice is critical for understanding mechanisms that control solute retention, transport and the development of habitable microenvironments.

The mapped location of both the liquid brine and hydrated salt network demonstrated a shared dependence on vertical positioning within the brinicle. Both brinicle compositions consistently displayed the lowest hydrated salt and liquid fractions at the base of the brinicles (Fig. 5). However, the liquid fraction profiles between the two salt types differed. While NaCl followed the same base to tip increase seen in the hydrated salt, CaCl_2_ had the highest liquid fraction at the mid and tip of the brinicle. The bulk movement of hydrated salt and liquid brine is consistent with previous literature demonstrating the exclusion of salts during freezing and the downward transport towards the growing tip of the brinicle [10, 14].

In contrast, radial mapping of the brine distribution revealed distinct compositional differences. For both hydrated salt and liquid fractions, CaCl_2_ brinicles exhibited strong localization of the brine within the inner channels of the structures (Fig. 5). This pattern is consistent with recent modeling efforts that have predicted the formation of a mushy brine region adjacent to the brine column [7]. Conversely, NaCl showed a uniform distribution of both liquid brine and hydrated salt, with no significant difference observed between sections (Fig. 5). Together, these results suggest that intrinsic physicochemical properties of the salts influence the lateral transport and distribution of brine throughout the brinicle ice matrix.

During growth, brinicles develop under a strong radial thermal gradient, with the warmer water on the outside of the ice and the cooler brine solution flowing inside the ice column. Previous work has shown that under thermal gradients, brine pockets migrate towards the warmer regions of the ice [40]. The velocity of this migration is directly related to the mass diffusion coefficient (D) of the dissolved ions [40]. Near the ice surface, Ca^2+^ has a lower diffusion coefficient than Na^+^ [41], resulting in a slower predicted migration of brine pockets to the outer edge under equal temperature gradients [40]. Additionally, previous work has shown that ions with larger hydration radii and hydration free energies (such as Ca^2+^) have lower diffusion abilities, increasing their entrapment in ice crystals during the freezing process [42]. This suggests that the compositionally distinct radial localization observed between NaCl and CaCl_2_ brinicles is a result of ion specific differences [40, 41].

The localization and distribution of liquid brine offers a physical mechanism for fluorescence distribution within the brinicle. For CaCl_2_, the tip at the inner hole is the convergent point where all three spatial measurements peaked (Figs. 4 and 5). In contrast, NaCl had a uniform spread of fluorescence with no region being significantly different (Fig. 5). The significant liquid fraction and fluorescence coupling (R^2^ = 49.88%, interaction p < 0.001) supports the observed spatial mirroring. Compositionally dependent slopes reveal two distinct localization behaviors for fluorescence: entrapment and dispersal (Fig. 7). For CaCl_2_, the relatively constrained liquid fraction sequesters the microspheres to a confined region. Conversely, NaCl’s connected distribution allows for uniform distribution of microspheres across the brinicle (Fig. 7A **and** B). Analogous to sea ice systems, the connectivity of the liquid brine networks governs the nutrient and biological transport within the ice at the macroscale and the microscale. [19, 34, 36].

When considering habitability on icy subsurface on Europa and Enceladus, the presence of liquid water alone is insufficient; the connectivity and spatial distribution of the liquid fractions are equally important [19]. This work demonstrates that brine compositions fundamentally influence the spatial structuring of liquid microenvironments within the ice. Brinicles grown under CaCl_2_ brine create isolated brine pockets, which may generate localized regions of highly concentrated liquid brine, nutrients, and biological material. As a result, this influences the persistence of liquid brine and promotes the preservation of biomolecules [43–45]. Brinicles within these systems could create discrete habitable zones for targeted sampling points and select for organisms that can tolerate higher solute concentration. In contrast, NaCl may produce more interconnected brine environments that retained lower localized concentrations but could allow for further nutrient and cellular mobility within a polycrystalline system [19, 36, 46, 47]. Although these systems would require wider spatial sampling, they may potentially support a wider diversity of organisms [48, 49]. Regardless of brine chemistry, sampling near brinicle efflux zones of Ocean World ice structures may represent the greatest potential for biosignature detection. Given the chemically distinct oceans of Earth, Europa and Enceladus, these compositionally driven differences in ice microstructures represent fundamentally different habitability niches within Ocean World ices [50, 51].

While this work establishes direct links between microsphere localization and compositionally driven brine microenvironments, several directions of study remain for future investigation. To better assess Ocean World habitability, an expanded understanding of mixed brine chemistries, temperature-relevant conditions, pressure and gravitational differences is needed to fully resolve their impact on the microenvironments within ice. Additionally, higher resolution mapping and measurements at different growth points would allow for temporal characterization of brine environments and greater insight into grain boundary liquid networks. Finally, incorporating living cells, biomolecules or representative biosignatures would allow for direct testing of habitability and detectability within these brinicle structures.

Salt composition fundamentally influences the microstructural environment of brinicle ice. While CaCl_2_ produced highly concentrated isolated regions near the brine channel, NaCl formed a distributed brine fraction that permitted the transport of microspheres throughout the ice. Ultimately, these findings demonstrate that the compositionally modulated brine localization generates distinct structural niches within ice. Consequently, the distribution, persistence and potential habitability of Ocean World ice shells may be strongly governed by ocean chemistry, with direct implications for biosignature preservation and future sampling efforts.

## Supplementary Material

Supplementary Files

This is a list of supplementary files associated with this preprint. Click to download.

- BriniclePaperSIv3.docx

## Figures and Tables

**Figure 1:**
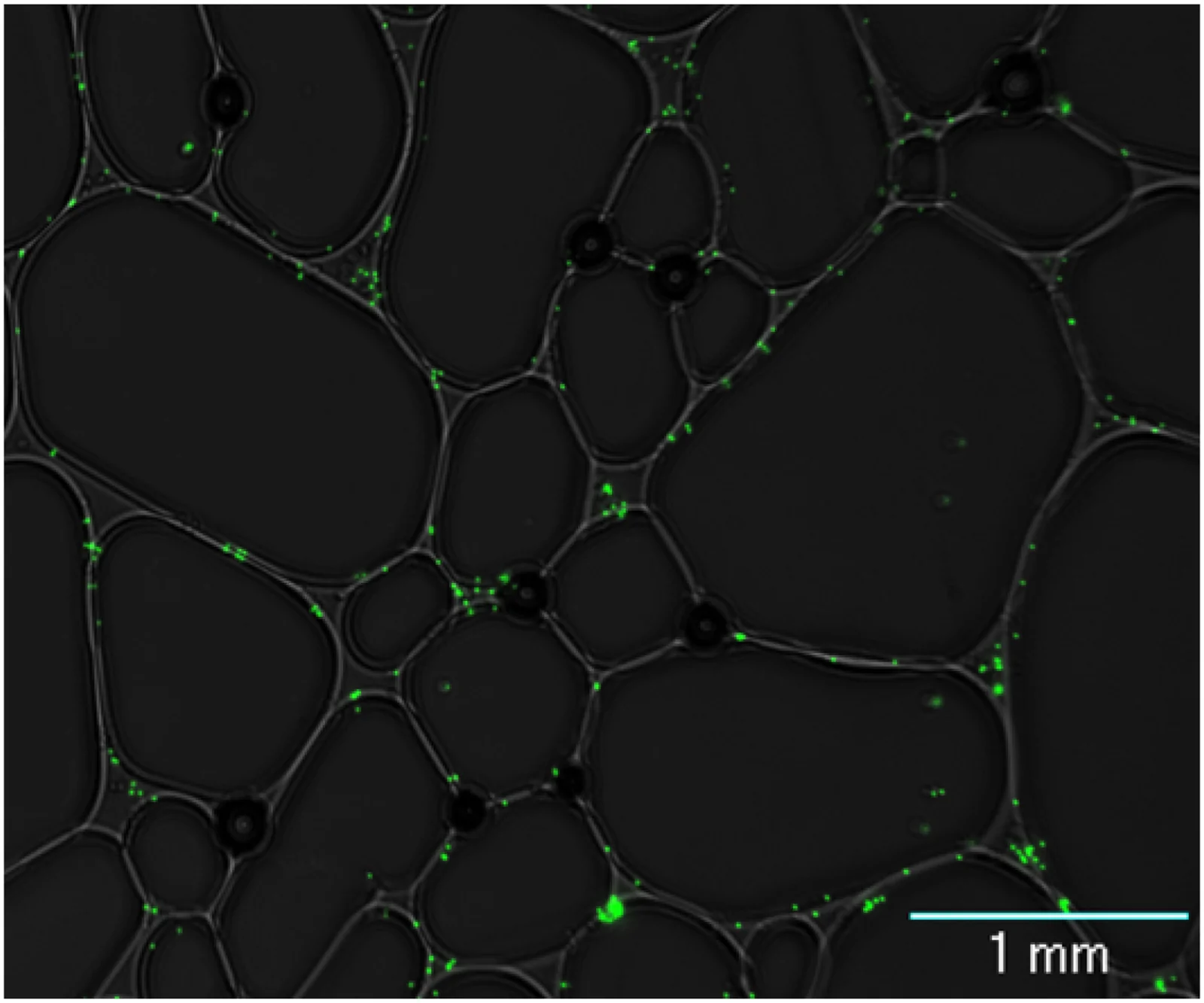
Image of ice grains where fluorescent microspheres can be seen localizing in liquid filled veins. Scale bar = 1 mm.

**Figure 2:**
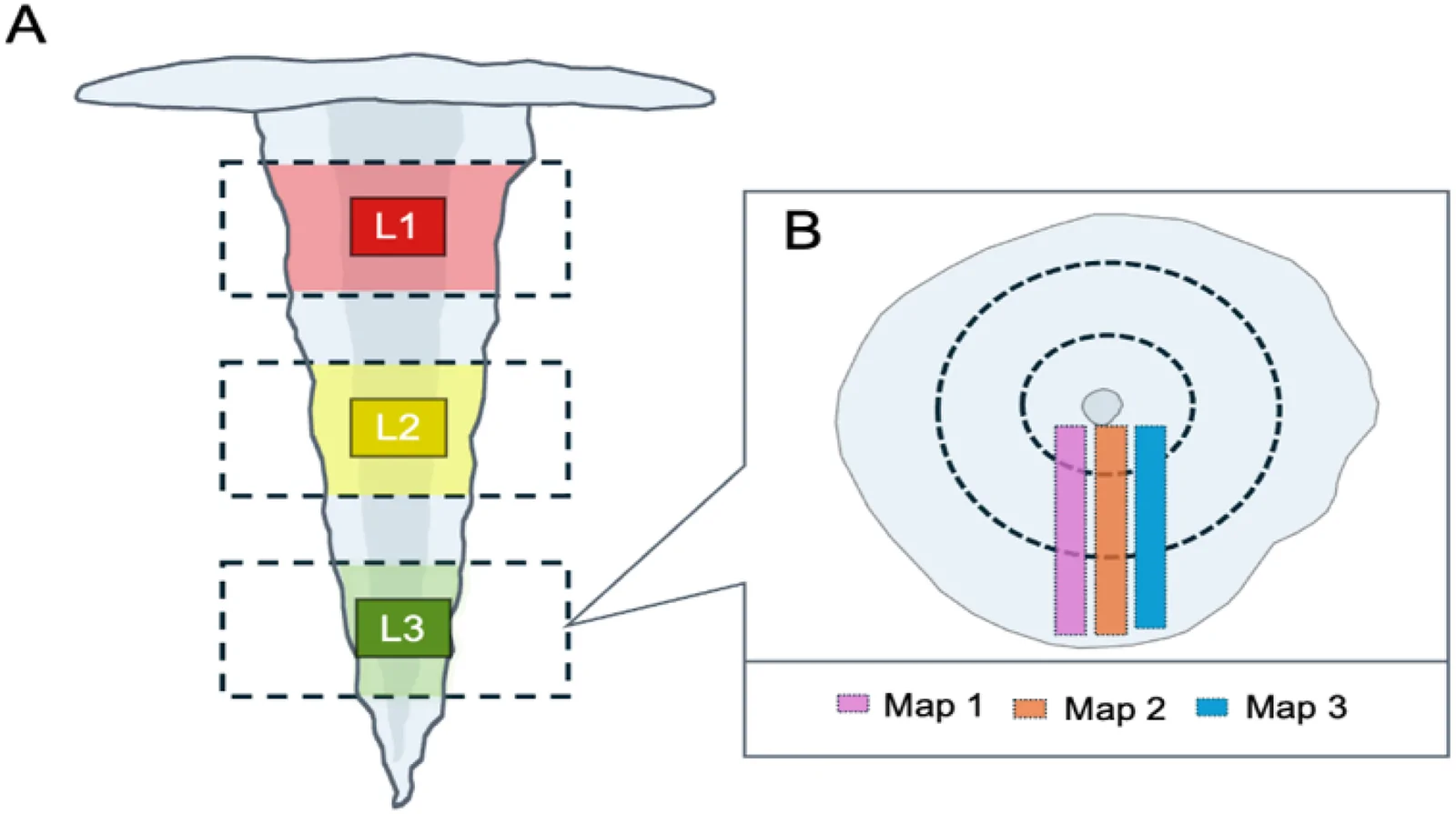
A) Brinicle sectioning pattern used for flow cytometry and Raman spectroscopy B) Raman mapping schematic of brinicle sections analyzed, Map 1-Map 3 are technical replicates.

**Figure 3:**
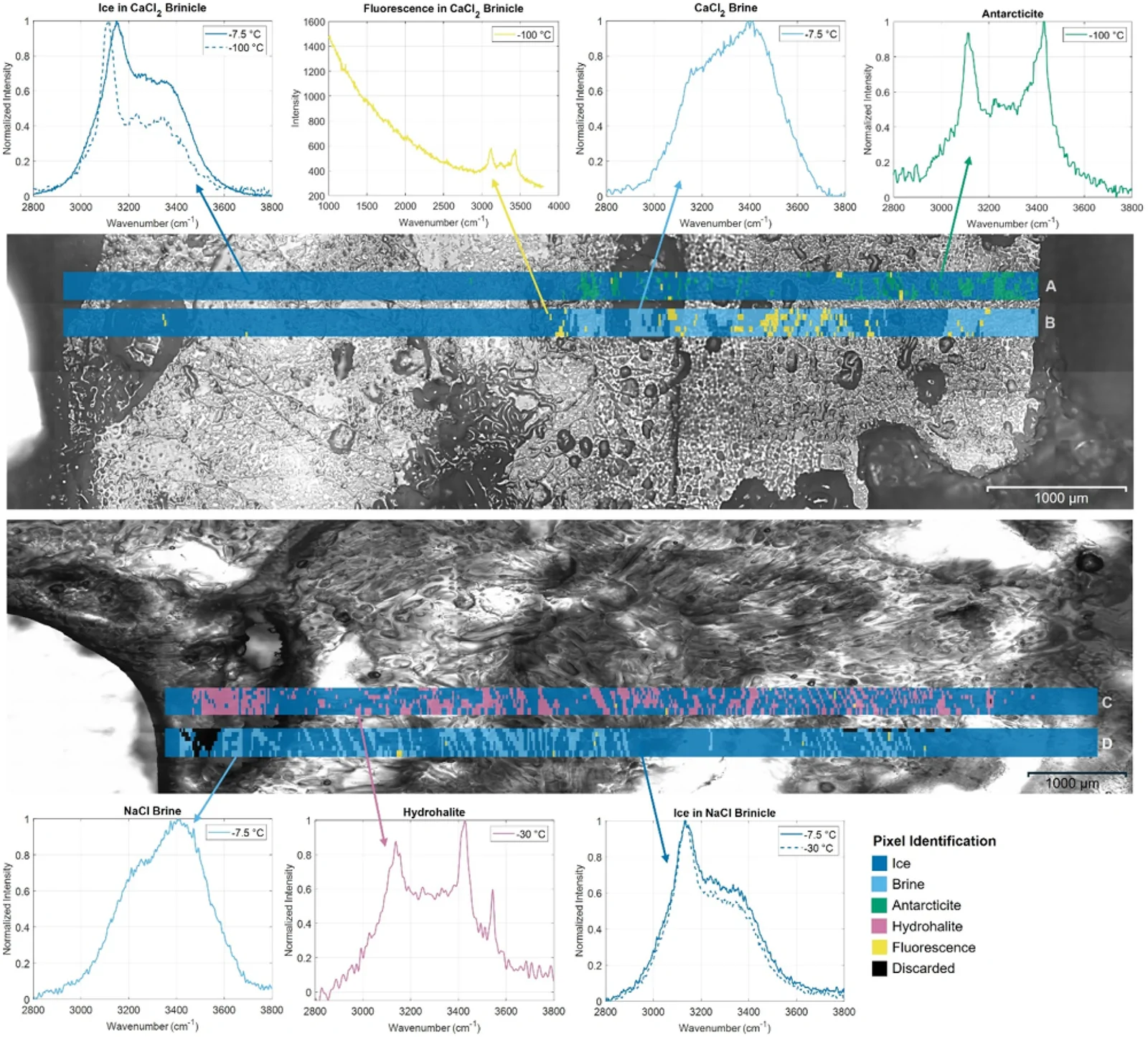
Mosaic images of CaCl_2_ brinicle tip (top grayscale image) and NaCl brinicle tip (bottom image) both captured at −7.5°C. Labeled Raman intensity maps (A-D) show the pixel compositions of a CaCl_2_ section measured at −100°C (A) and −7.5°C (B) and a NaCl section measured at −30°C (C) and −7.5°C (D). Arrows identify examples of spectra for each pixel type (except discarded pixels) present in the composition maps to familiarize the reader with spectra shapes mentioned in the text. Note that though the below-eutectic and above-eutectic maps are presented on the same image here, they were taken at different times following the temperature ramp described in the text. Each map shown here was taken along with two other parallel maps to account for variability within section.

**Figure 4:**
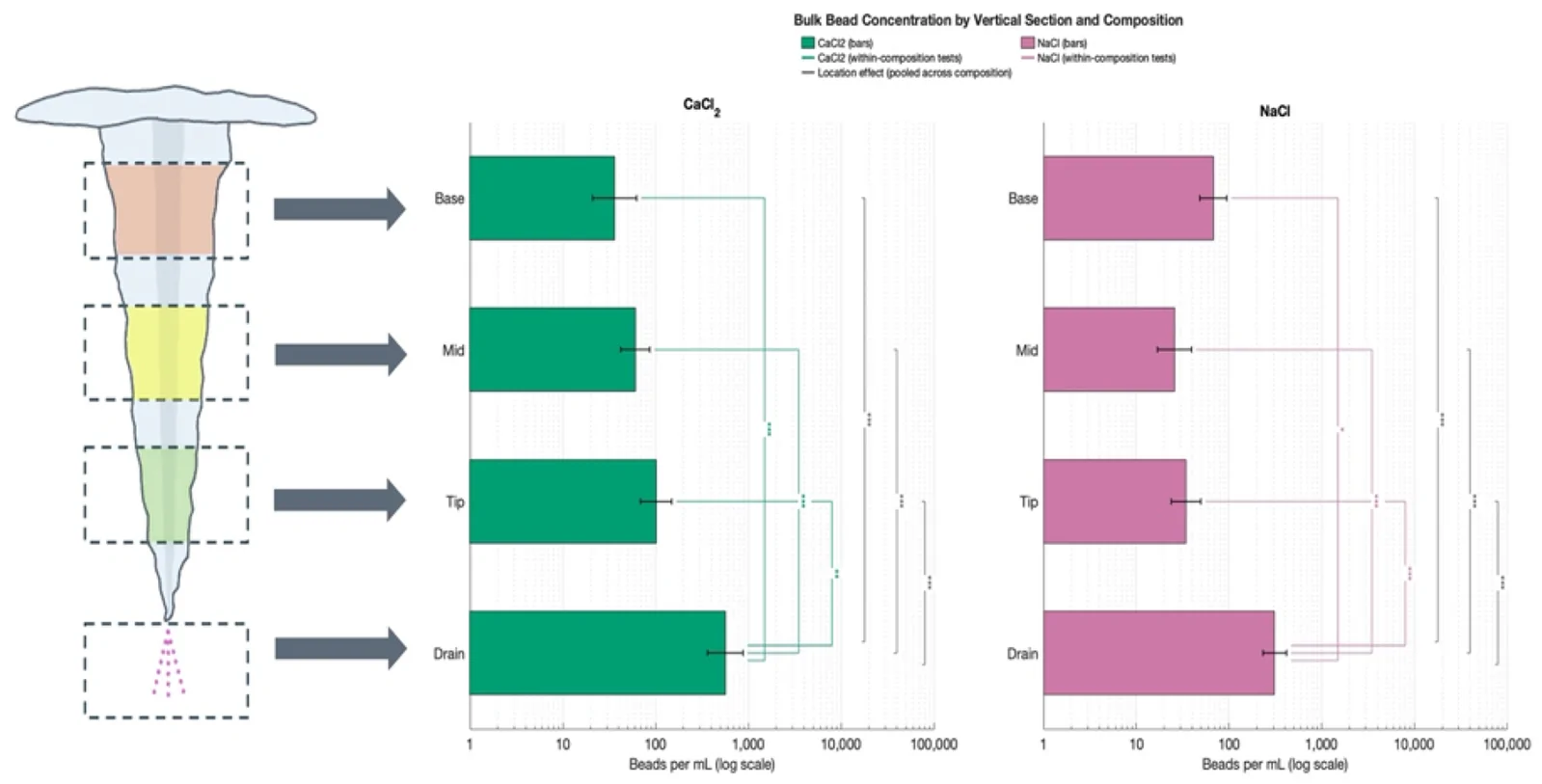
Bulk fluorescent microsphere concentrations are shown across four vertical sections (Base, Mid, Tip, Drain). Mean microspheres per mL shown on a log scale for CaCl_2_ why ref and not (green) and NaCl (pink) treatments. Error bars represent standard error of the mean). Asterisks indicate significance level (*p<0.05, **p<0.01, ***p<0.001). Bracket color indicates comparison type. Pink and green brackets denote pairwise comparisons within a single salt composition (NaCl or CaCl_2_, respectively). The gray brackets denote the effect of position, combined across composition.

**Figure 5:**
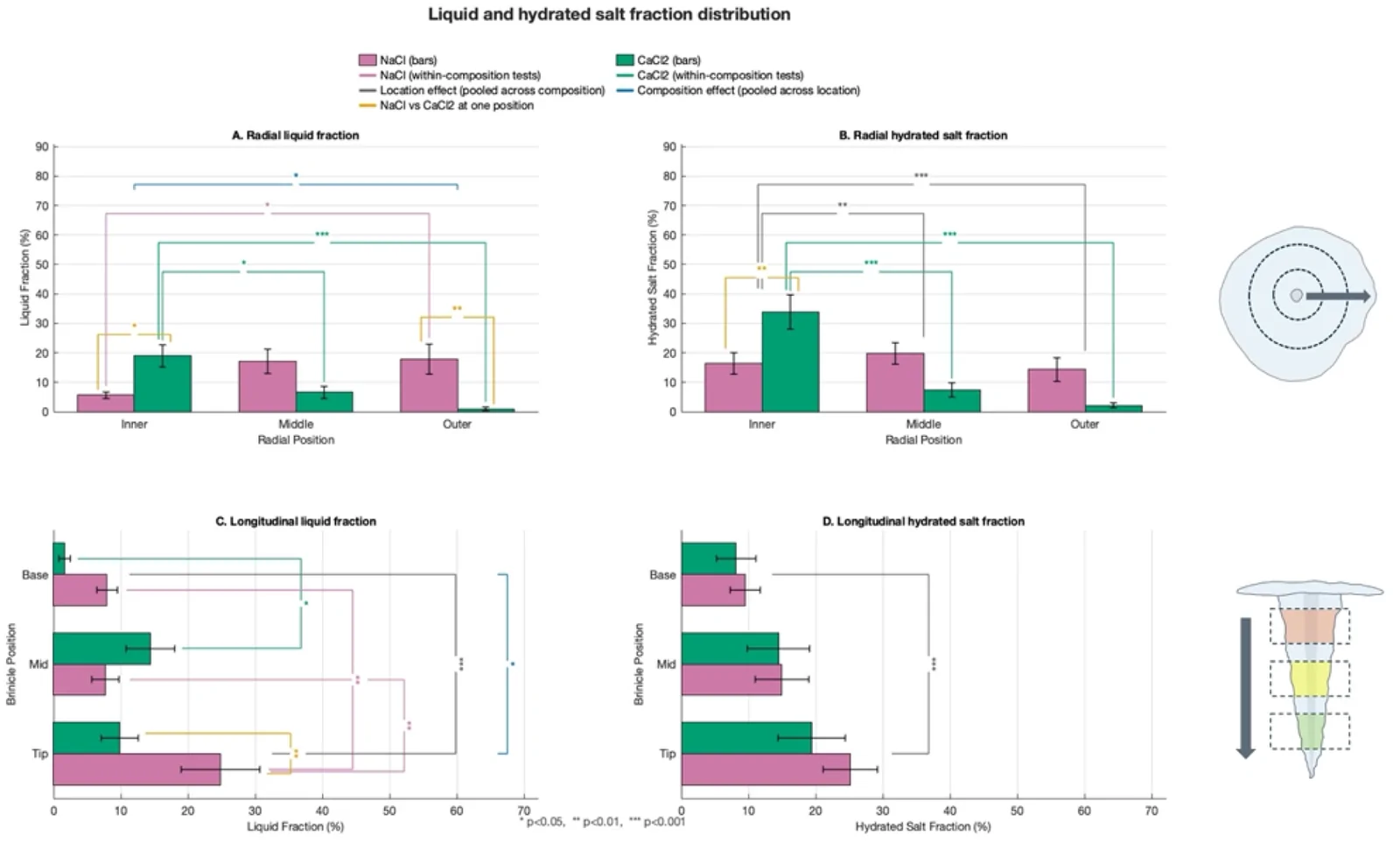
Liquid and hydrated salt fraction distribution within brinicles grown under NaCl (pink) and CaCl_2_ (green) conditions. A) Radial liquid fraction distribution across the inner, middle, and outer regions of the brinicle cross section. B) Radial hydrated salt fraction across brinicle cross section. C) Vertical liquid fraction distribution across base, mid, and tip sections. D) Vertical hydrated salt fraction distribution across sections. Diagrams to the right illustrate the radial (top) and longitudinal (bottom) sampling orientations. Error bars represent standard error of the mean. Asterisks indicate significance level (* = p < 0.05, ** = p < 0.01, *** = p < 0.001). Bracket color indicates comparison type. Pink and green brackets denote pairwise comparisons within a single salt composition (NaCl or CaCl_2_, respectively). The gray brackets denote the effect of position, combined across composition. The blue brackets denote the effect of composition, combined across position. The orange brackets denote a direct NaCl vs. CaCl_2_ comparison at a single position.

**Figure 6:**
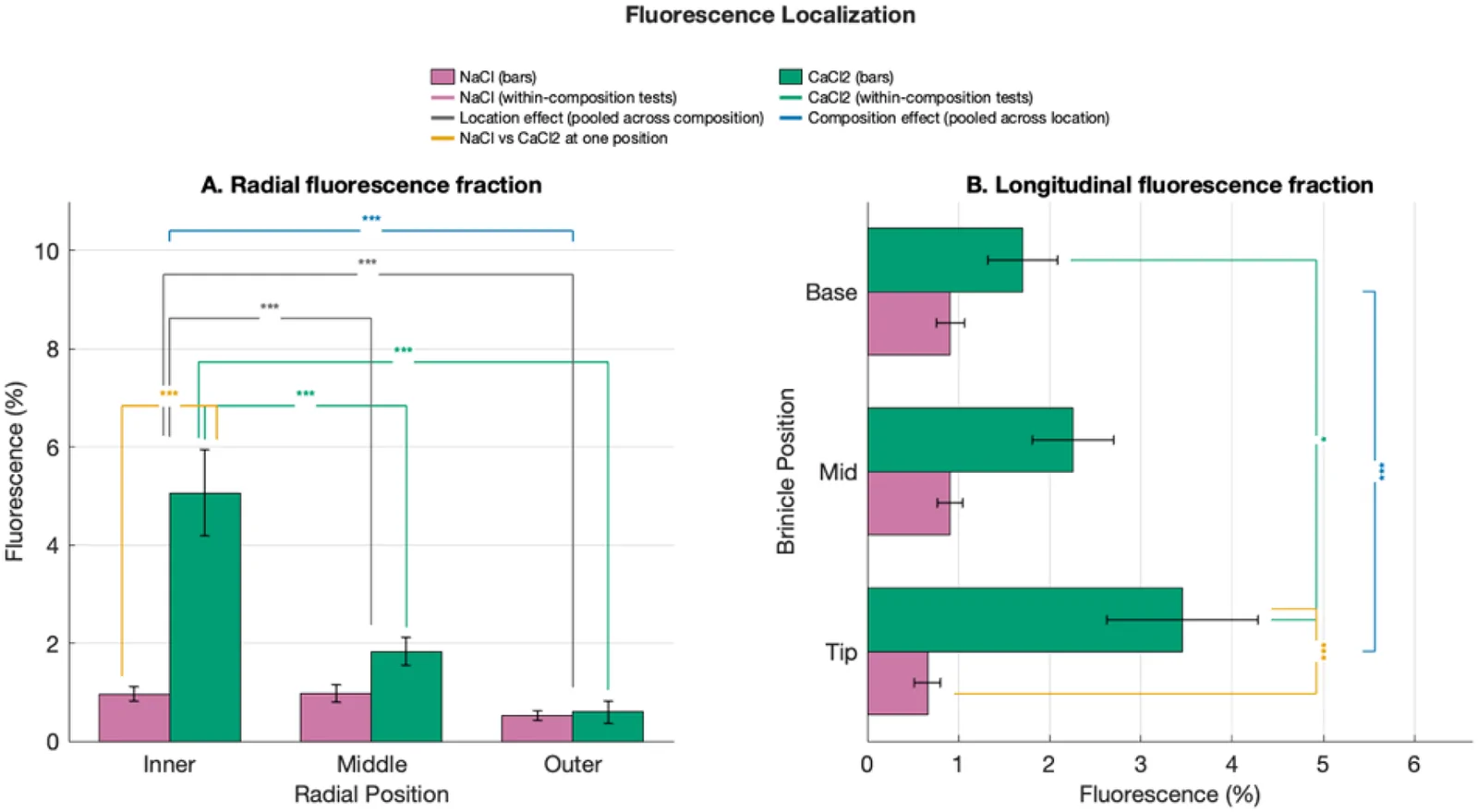
Fluorescence fraction distribution within brinicles grown under NaCl (pink) and CaCl_2_ (green) conditions. A) Radial fluorescence fraction distribution across the inner, middle, and outer regions of the brinicle cross section. B) Vertical fluorescence fraction distribution across sections. Error bars represent standard error of the mean. Asterisks indicate significance level (* = p < 0.05, ** = p < 0.01, *** = p < 0.001). Bracket color indicates comparison type. Pink and green brackets denote pairwise comparisons within a single salt composition (NaCl or CaCl_2_, respectively). The gray brackets denote the effect of position, combined across composition. The blue brackets denote the effect of composition, combined across position. The orange brackets denote a direct NaCl vs. CaCl_2_ comparison at a single position.

**Figure 7:**
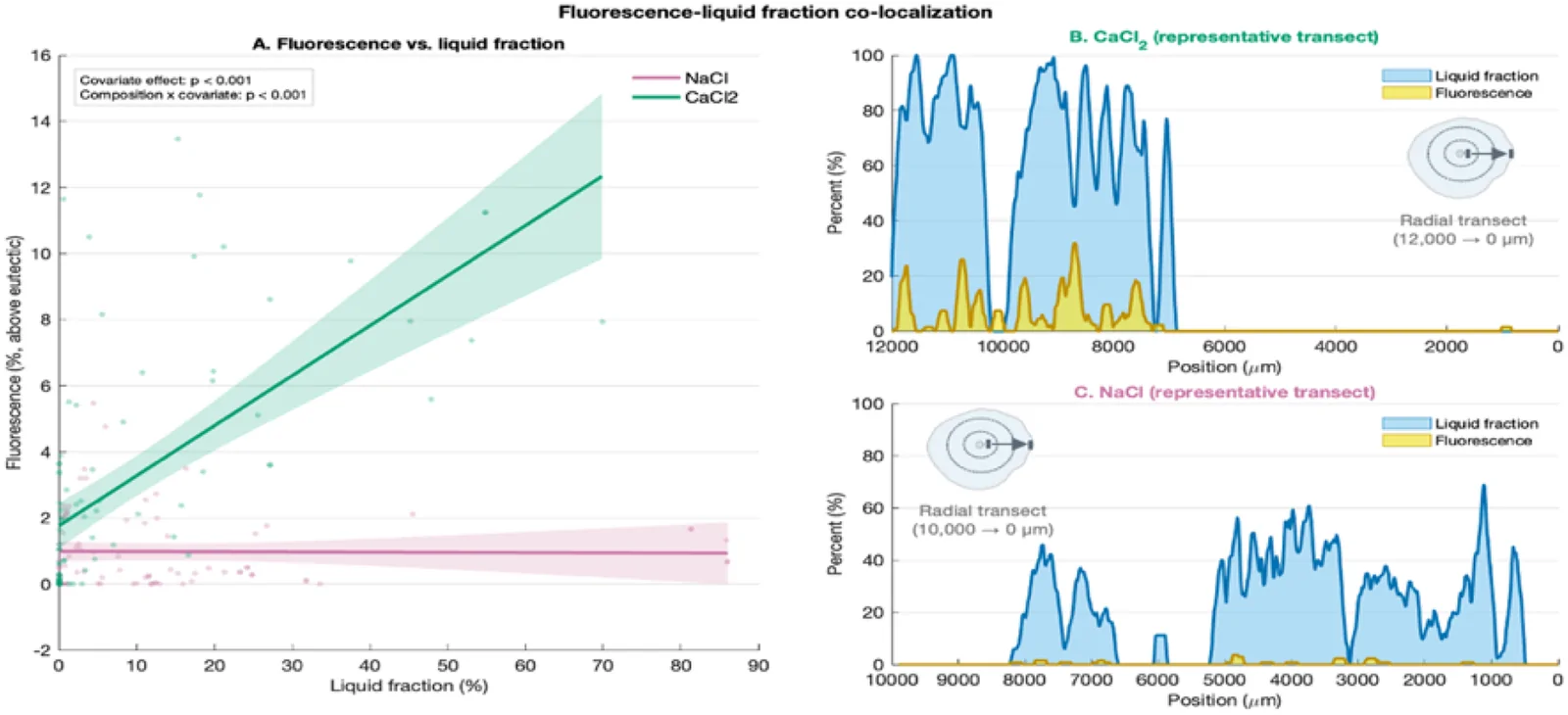
A) Model fitted relationship between the liquid fraction and the above eutectic (−7.5°C) fluorescence fraction for NaCl (pink) and CaCl_2_. Individual observations are represented by points. The lines and shaded regions represent the model fitted slope and 95% confidence intervals. B) The representative radial transects used in figure 3 for CaCl_2_, depicting colocalization of liquid fraction (blue) and fluorescence fraction (yellow). Map is depicted form inner hole side to outer edge side (12,000 to 0 μm). C) The representative radial transects used in figure 3 for NaCl. Radial plot is mapped from inner hole side to outer edge side (10,000 to 0 μm). Inset schematics depict the sampling orientation.

## Data Availability

The dataset used during the current study is available upon request to the corresponding author.
